# A scoping review of the qualitative literature reporting experiences of living with a stoma for inflammatory bowel disease

**DOI:** 10.1111/jan.16254

**Published:** 2024-05-23

**Authors:** Ryan Essex, Lesley Booth, Fuschia Sirois, Jennie Burch, Lesley Dibley

**Affiliations:** ^1^ Centre for Chronic Illness and Ageing Institute for Lifecourse Development, Faculty of Education, Health and Humans Sciences, University of Greenwich London UK; ^2^ Cambridge Rare Disease Network Cambridge UK; ^3^ Department of Psychology Durham University Durham UK; ^4^ Academic Institute, St Mark's Hospital London UK

**Keywords:** attributes, barriers, facilitators, inflammatory bowel diseases, ostomy

## Abstract

**Aims:**

Surgical treatment for inflammatory bowel disease (IBD) potentially includes stoma formation. Although positive clinical outcomes are widely reported, patients' responses to stoma surgery, including coming to terms with and adjusting to the stoma, vary widely. This scoping review charts the qualitative literature addressing the question: *What is known about any personal psychosocial and quality of life factors that inform adjustment to living well with an intestinal stoma for IBD*?

**Design:**

A scoping review methodology was employed.

**Data Sources:**

Searches of Scopus, Web of Science, CINAHL, Medline and PsycInfo in August 2023.

**Review Methods:**

Levac et al.'s (2010) methodology was followed. PRISMA‐ScR guidelines were adhered to.

**Results:**

Thirteen cross‐sectional studies were included, involving a total of 142 participants. Four themes were identified: (1) facilitative factors; (2) barriers to adjustment; (3) personal attributes; and (4) time and temporality. Data indicate *that* personal and psychological factors influence adjustment, but not *how* this occurs. Adjustment takes longer to achieve than is conventionally (clinically) expected.

**Conclusion:**

All available evidence is cross‐sectional. The identified gap in the evidence is the notable lack of longitudinal research to assess, monitor and understand the complex process of adjustment in people with IBD having stoma‐forming surgery. Detailed understanding of the process of adjustment would enable more targeted support for patients preparing for, and learning to live with, a stoma for IBD.

**Impact:**

This paper highlights the need to understand the multiple personal and psychosocial factors that affect adjustment to life with a stoma and identifies that adjustment takes significantly longer than the few weeks required to become competent in managing the stoma.

**Patient or Public Contribution:**

Not applicable.

## 1 INTRODUCTION

Approximately 130,000 people in the United States (Mulita & Lotfollahzadeh, 2023) and 21,000 people in the UK (NHS Digital, 2023) undergo intestinal stoma surgery annually for bowel conditions including cancer and inflammatory bowel disease (IBD)—encompassing Crohn's disease (CD) and ulcerative colitis (UC). Of the 500,000 people in the UK with IBD (HDRUK, 2020), approximately 30% with UC and 70% with CD will require intestinal surgery, potentially with a temporary or permanent stoma, at some stage (Frolkis et al., 2013; Kaplan et al., 2012).

Quantitative evidence indicates that quality of life (QoL) following stoma‐forming surgery is often high (Aluzaite et al., 2020) especially when stoma formation achieves clinical remission (Abdalla et al., 2016). However, the relationship between the severity of underlying disease, time since stoma formation and reliability of the stoma appliance is inconsistent, highlighting ‘*the multifactorial nature of the quality of life concept*’ (Kaplan et al., 2012, p. 987); individual patient, psychosocial and cultural factors may also be influential. Disgust trait potentially influences attitudes towards an intestinal stoma (Smith et al., 2009), and there is indication that age, personality, stigma, childhood socialization regarding bodily functions and other psychosocial aspects may be contributory factors (Dibley, Norton, & Whitehead, 2018; Knowles et al., 2013). If the patient *chooses* stoma‐forming surgery over, for example, an internal pouch, then their subsequent QoL will be influenced positively (Kuruvilla et al., 2012). However, people with IBD often worry about the prospect of stoma‐forming surgery and their decision‐making is influenced by numerous non‐clinical factors (Dibley, Czuber‐Dochan, et al., 2018). Whilst many do embrace and respond positively to life with a stoma, patients often approach stoma surgery with pre‐existing concerns that can affect adaptation to and acceptance of the stoma, thus influencing QoL outcomes. The topic of bowels is a universally taboo. Concerns about potential visibility of a stoma appliance on the ostomate's abdomen can tap into deep‐seated social rules about privacy (Dibley, Norton, & Whitehead, 2018), negatively affecting QoL. Pre‐, peri‐ and postoperative education from specialist stoma nurses focusses on teaching practical skills required for stoma management (Danielsen, Burcharth, & Rosenberg, 2013) with a generally expected timescale of 3 months for gaining competence. Quantitative evidence, primarily based on retrospective cross‐sectional studies, measures QoL and demonstrates *whether* this improves (Camilleri‐Brennan et al., 2003; Pittman et al., 2009; Umanskiy & Fichera, 2010). One useful qualitative systematic review highlights the positive influence of acceptance, adaptation and autonomy on people living with a stoma for colorectal cancer (Capilla‐Díaz et al., 2019). Much less is known about the long‐term experiences of patients living with a stoma due to IBD or about *how* personal, emotional, cultural and psychosocial factors contribute to an improved QoL over time. We aimed to identify personal factors that facilitate or hinder adjustment to life with a stoma for people with IBD, identify any gaps in the evidence and support the case for further research.

## 2 METHODS

This scoping review was guided by an established six‐stage methodology (Levac et al., 2010) (Table 1) and is reported as per the PRISMA‐SCR guidelines (Tricco et al., 2018). As recommended (Pollock et al., 2021; Tricco et al., 2018), the protocol for this review is registered on the Open Science Framework (Essex et al., 2021). A detailed methodology reporting the search strategy and review process is provided in Supplementary File S1.

**TABLE 1:** Levac et al. (2010) six‐stage scoping review methodology (Levac et al., 2010).

Framework stage	Purpose
Stage 1	Identifying the research question
Stage 2	Identifying relevant studies
Stage 3	Study selection
Stage 4	Charting the data
Stage 5	Collating, reporting and summarizing the results
Stage 6	Consultation with stakeholders

## 3 RESULTS

**FIGURE 1:**
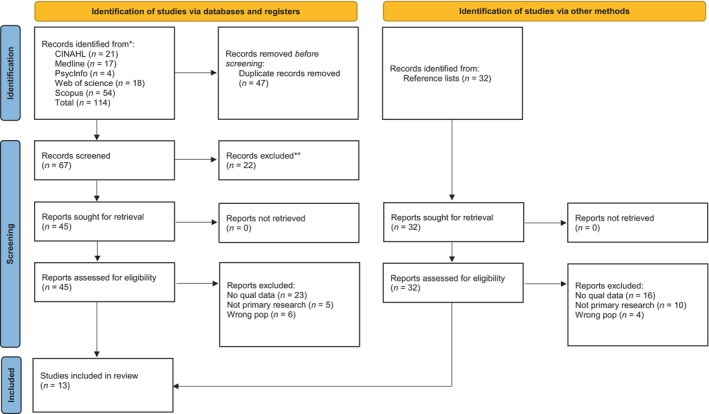
PRISMA 2020 flow diagram for new systematic reviews, which included searches of databases, registers and other sources.

Initial searches returned 114 results. Following removal of duplicates, 67 papers remained. Titles and abstracts were screened against inclusion criteria, with a further 22 excluded. Reference lists of the remaining 45 papers were hand‐searched, identifying a further 32 papers. Thus, 77 papers underwent full‐text screening. Sixty‐four papers did not meet the inclusion criteria; 13 were retained for review (Figure 1 and Table 2).

**TABLE 2:** Summary of selected qualitative articles and data charting.

Author(s), year, country study title	i. Qualitative methodology ii. Sampling iii. Data capture Iv. Data analysis	Sample: i. *N* = ii. Age (range) iii. Gender iv. Diagnosis v. Type of stoma (*N*) vi. Time with stoma (range)	Adjustment mechanisms/key issues extracted during data charting realizations, personal attributes, attitudes, or aspects of personality that are perceived to help or hinder adjustment
Allison et al., 2013, UK Surgery in young adults with inflammatory bowel disease: a narrative account	i. Narrative approach ii. Purposive iii. Semi‐structured interviews iv. Story‐mapping, re‐storying	i. *N* = 24 ii. 18–25 years; iii. M = 11, F = 13 iv. CD = 17; UC = 7 v. Not specified vi. Unclear	***Helpful factors***: Negotiating timing of surgery and having time to prepare and anticipate benefits; information‐seeking reduces stress and anxiety, assists in pre‐surgery decision‐making; accepting need for surgery occurs gradually—turning point when symptoms/situation no longer tolerable. Concerns, feelings and opinions acknowledged by medical staff, and emotional support and continuity of care from specialist nurses. Supportive family—particularly parents—enabling growing independence as recovery progresses; practical assistance; peer support—for young women, mainly emotional support, comfort and reassurance; for young men—peers assisted with normalizing, and practical help. Personal qualities: bravery, determination, commitment, inner strength/resilience, positive attitude, not letting life be defined by, or limited by illness, making positive comparisons (between pre‐surgery debilitation due to illness, and improved status afterwards), emotional maturity ***Hindrances***: Restricting social life/dating until stoma reversed; anxiety about surgery, especially if not had it before; overprotective parents; over‐vigilance in intimate relationships; for women post‐surgery—body image (clothing, scars, uncontrolled bowel activity). For men—body image only due to the stoma. Repeated surgeries with minimal evidence of overall improvement
Danielsen et al., 2013, Denmark Learning to live with a permanent intestinal ostomy	i. Unspecified ii. Not specified iii. Focus groups iv. Content analysis	i. *N* = 15 (5 with IBD) ii. 63 (22–83) iii. Unclear iv. No diagnosis detail other than ‘IBD’ v. Ileostomy (*n* = 5) vi. 3 years (2–10 years)	***Helpful factors***: Contact with and support from friends; dedicated nurse support for consistency; group‐based learning for ostomy education; lay teachers; real‐life ‘simulation’ experiences; information that set realistic expectations of potential post‐op issues; coping strategies—covering, disclosing and disguising the stoma ***Hindrances***: Perceived stigma/taboo of stoma prevents socialization; belief that new relationships would not be possible; concerns that others could detect their stoma; heightened need to control their outward appearance to avoid disclosure of the stoma; identity as young adult affected—having a stoma did not match their view of themselves. Poor attitudes of ward staff post‐surgery had an enduring negative effect on individual emotional and relational needs; hospital staff setting unrealistic expectations (particularly that there would be ‘no problems’) led to heightened anxiety and concerns when stoma‐related difficulties occurred

Author(s), Year Country Study title	i. Methodology ii. Sampling iii. Data capture iv. Data analysis	Sample: i. *N* = ii. Age (range) iii. Gender iv. Diagnosis v. Type of stoma (N) vi. Time with stoma (range)	Adjustment mechanisms/key issues extracted during data charting Realizations, personal attributes, attitudes, or aspects of personality that are perceived to help or hinder adjustment
Horgan et al., 2020, Ireland The young adult male's perception of life with inflammatory bowel disease and a stoma: a qualitative evaluation	i. IPA ii. Purposive iii. Semi‐structured interviews iv. IPA	i. *N* = 5 ii. 25 (23–30) iii. All male iv. UC (3); CD (1); IBD‐U (1) v. Temporary end ileostomy (4); permanent end ileostomy (1) vi. Max 1 year	***Helpful factors***: Post‐op support from specialist nurses; directness of surgeons; using clothing or dressings to conceal the stoma from others—peer acceptance highly valued, invisibility of the stoma crucial for maintaining secrecy and reducing stigma; drawbacks such as leakages considered minor compared to the suffering they endured pre‐surgery—embracing the health the stoma brings; accepting the stoma as ‘there’ and becoming competent with its management; conscious decisions to avoid negative thinking—‘to get on with it’; feeling in control—of the stoma, and emotionally/psychologically. Adjustment occurred after acceptance of the stoma—partly due to the newfound predictability and freedom it brought and being pleasantly surprised by the minimal impact of the stoma on important personal activities ***Hindrances***: poor pre‐op information from clinicians, and risks when sourcing information from the internet; emergency stoma formation = poor pre‐op guidance, lack of preparedness; leaving the possibility of a stoma until very late in the illness trajectory compounds the shock of needing the surgery; no real choice when surgery was the only option for effectively managing symptoms; no access to peers with the same experience. Four key emotions: embarrassment (stoma‐related sounds and smells); worry and fear (Including potential negative impact of stoma on ability to form romantic partnerships); insecurity (about their body/body image), and frustration
Morris and Leach, 2017, UK A qualitative exploration of the lived experiences of patients before and after ileostomy creation as a result of surgical management for Crohn's disease	i. HP ii. Purposive iii. Semi‐structured interviews iv. Unclear	i. *N* = 10 ii. 52.2 (34–83) iii. M (4), F (6) iv. All CD v. Permanent ileostomy (all) vi. 18.3 years (3–36 years)	***Helpful factors***: Adjusting to a stoma is a transition—it takes time; pre‐surgery experience of CD effects post‐surgery acceptance of stoma—more debilitating disease pre‐surgery usual corresponds with greater tolerance of stoma due to dramatic improvement in health. Stoma seen as facilitating a normal life (where participants felt in control, rather than their CD controlling them). Emotional support from friends and family ***Hindrances***: Memories of pre‐stoma Crohn's can negatively affect health‐related behaviours, such as not wanting to lose steroid‐related weight gain due to concerns of reminders of being unhealthily thin prior to having the stoma

Author(s), Year, Country Study title	i. Methodology ii. Sampling iii. Data capture iv. Data analysis	Sample: i. *N* = ii. Age (range) iii. Gender iv. Diagnosis v. Type of stoma (*N*) vi. Time with stoma (range)	Adjustment mechanisms/key issues extracted during data charting Realizations, personal attributes, attitudes, or aspects of personality that are perceived to help or hinder adjustment
Nicholas et al., 2008, Canada Struggles, strengths and strategies: an ethnographic study exploring the experiences of adolescents living with an ostomy	i. Ethnography ii. Not specified iii. Semi‐structured interviews and focus groups iv. Content analysis	i. *N* = 20 ii. 15.3 years (13–19 years) iii. M (9), F (11) iv. UC (all) v. Not specified (ostomy or J‐Pouch) vi. 2.9 years (3 months – 10 years)	***Helpful factors***: Adjustment takes place over time—figuring out ‘what works for you’ in stoma care; reaching a level of ‘okay‐ness’ with the ostomy to be able to tell peers about it; choosing carefully who and how to tell; acceptance and support from peers; others responses helped teens assess their true value as a friend; establishing an internal locus of control and valuing their own self‐worth; increasing mastery, confidence and independence, achieved through self‐education, and stoma care practice; support from family, especially in the immediate pre‐op period; stoma experience helped them realign important life perspectives and priorities and grow their emotional intelligence; being honest about personal feelings, emotions and needs; emotional and psychological move towards acceptance, which occurs at different rates for different people. Social support, especially peer support, is helpful in moving forwards and feeling less isolated. Talking with others who have had the same experience Being informed, from a variety of sources, enables the taking on of self‐care, and with that comes confidence that the ostomy is manageable. Recognizing the benefits of the stoma over previous illness ***Hindrances***: Concerns re body image; over‐protective parents; stigma, shame and embarrassment preventing them from disclosing about the ostomy to friends; fear of unplanned disclosure; worries about impact of their IBD on family members, especially siblings; influences of upbringing on expectations of behaviour (not cry, be happy)
Persson and Hellström, 2002, Sweden Experiences of Swedish men and women 6 to 12 weeks after ostomy surgery	i. Phenomenology ii. Purposive iii. Individual unstructured interview iv. Giorgi's phenomenological analysis	i. *N* = 9 ii. 53.3 years (44–67 years) iii. M (5), F (4) iv. Rectal cancer (2); Bladder cancer (2); Diverticulitis (1); RV fistula (1); UC (3) v. UC participants: ileostomy (2); colostomy (1) vi. 6–12 weeks	***Helpful factors***: Better control, being able to plan; better health—recognizing the benefits of the stoma ***Hindrances*:** Concerns re body image; negative emotions—disgust, shock; disruption to sexual activities—sexual experiences altered (for the worse); uncertainty and fear; concerns re gas/odour leakage, harming the stoma, it being visible to others (stigma); limited social contact in part due to concerns about others gossip, feelings of not coping, and not being ready to resume previous activities

Author(s), year, country Study title	i. Methodology ii. Sampling iii. Data capture Iv. Data analysis	Sample: i. *N* = ii. Age (range) iii. Gender Iv. Diagnosis v. Type of stoma (*N*) Vi. Time with stoma (range)	Adjustment mechanisms/key issues extracted during data charting Realizations, personal attributes, attitudes, or aspects of personality that are perceived to help or hinder adjustment
Polidano et al., 2020, UK Embracing a ‘new normal’: the construction of biographical renewal in young adults' narratives of living with a stoma	i. Constructivist grounded theory and narrative inquiry ii. Purposive iii. Individual semi‐structured interview iv. Constructivist grounded theory T and narrative analysis	i. *N* = 13 ii. 24.2 years (19–29 years) iii. M (4), F (9) iv. CD (7); UC (6) v. Temp (8); permanent (5) vi. 1.6 years (3mo‐5 years)	***Helpful factors***: stoma brings positive physical and social transformation, alleviates the disruptive effects of illness;; accepting the need to make some adjustments because of the stoma—but this is better than missing out on social events; seeing the stoma surgery (and consequent relief from IBD symptoms) as an opportunity to re‐examine personal goals; realizing they can cope with the stoma increases their general self‐confidence; contrast between pre‐stoma (ill self) and post‐stoma health—greater illness severity/duration pre‐surgery = greater perceived benefits of surgery; time—once competent with stoma management, benefits become apparent; ‘good’ stoma function (minimal leakage, noise, odour) = greater perceived benefits; self‐efficacy in stoma management; being in a supportive relationship; having a ‘fighting attitude’/ maintaining personal agency; viewing life with the stoma as better than life without; accepting psychological challenges as a reasonable trade‐off for feeling healthy. ***Hindrances***: Benefits not apparent soon after surgery, so much to learn to cope with, first month after surgery is hard; being single / unpartnered (and wanting to be with someone); CD diagnosis: colectomy not curative; mental health issues; leakages, concerns about body image, and personal relationships, reduced self‐confidence; not seeing or feeling the stoma to be beneficial; wanting life to be as it was before (illness and stoma) and struggling when that doesn't happen; holding on to pre‐stoma self, being reluctant to accept the change; temporary stoma—suspending activities until it is reversed, avoiding integration/acceptance; minimal perceived improvements in health between pre‐ and post‐stoma status; stoma not considered an acceptable trade‐off for relief of symptoms.
Savard and Woodgate, 2009, Canada Young peoples' experiences of living with ulcerative colitis and an ostomy	i. HP ii. Purposive iii. Two unstructured interviews per participant iv. Van Manen's HP analysis	i. N = 6 ii. 19–24 years (no mean) iii. M (1), F (5) iv. UC (6) v. Temporary ileostomy (6) – (5 reversed at point of interview) vi. Not specified	***Helpful factors***: Stoma acceptable because not permanent; expectation that future pouch surgery would restore them to normality; better health after ostomy surgery—recognizing the benefits of the stoma, more freedom; better person because of experience—comfortable with the young adult they had become; emotional acceptance. ***Hindrances***: stigma and taboo; body image/social expectations of ‘perfect’; embarrassment; visibility of stoma under clothes; different from peers due to stoma; not ready to ‘take the stoma on’ post‐op; difficulties with stoma management—leakages; unwilling to be independent with stoma care; comparing stoma unfavourably to previous life.

Author(s), Year, Country Study title	i. Methodology ii. Sampling iii. Data capture iv. Data analysis	Sample: i. *N* = ii. Age (range) iii. Gender iv. Diagnosis v. Type of stoma (N) vi. Time with stoma (range)	Adjustment mechanisms/key issues extracted during data charting Realizations, personal attributes, attitudes or aspects of personality that are perceived to help or hinder adjustment
Sinclair, 2009, Canada Young adults with permanent ileostomies	i. Narrative inquiry ii. Purposive iii. Individual unstructured interviews iv. Narrative Knowing Framework	i. *N* = 7 ii. 34.5 years (24–40 years) iii. M (3), F (4) iv. UC (4), CD (2), Chronic constipation (1) v. Permanent ileostomy (7) vi. Max 4 years	***Helpful factors***: Having time to prepare for stoma surgery; educating oneself about the options; being informed via a variety of sources. Time—it takes time; connecting with others who had also had stoma surgery; family support; telling one's story (but in a safe place); a previous temporary stoma is good preparation for a permanent one ***Hindrances***: If stoma surgery is driven by crisis; fear and shock of the new stoma; no obvious perceived benefit; negative body image/impact on intimacy/relationships—not wanting to expose themselves physically and emotionally in a new relationship; inadequate written information; poor pre‐ and post‐operative education; objectification/non‐acceptance of the stoma; negative reaction of nurses to the stoma, and lack of appreciation of how traumatic the experience was for patients; nurses' lack of insight; concerns about appliance leakages; negative pre and post‐operative experiences have a long duration of impact; vulnerable age range—significant vulnerabilities in their belief that they could or would not cope as an ostomate; being different ‘damaged goods’; fear, perceptions of self and also how others would perceive them was very negative; secrecy—unwilling to acknowledge stoma to others outside the family
Smith et al. 2016, UK The psychological challenges of living with an ileostomy: an interpretive phenomenological analysis	i. IPA ii. Purposive stratified iii. Semi‐structured individual interviews iv. IPA	i. *N* = 21 ii. 46.7 years (23–72 years) iii. M (10), F (11) iv. UC (11), CD (5) v. Permanent ileostomy (21) vi. 8.2 years (2–55 years)	**Helpful factors**: humour; confidence of ability to cope with the stoma brings perspective to prior unwarranted concerns; into perspective; embracing new status (as disabled) and working with that; taking charge of who is told about the stoma, and how; telling others brings support; unstinting protection and support from intimate partners; the acceptance of children—their parent is still their parent—no judgement; finding support from other sources, if not available from family; older age range had more belief that they could cope with having a stoma going forward to re‐engage with ‘normal’ life; time to adapt ***Hindrances***: Negative body image for both males and females; feeling ‘incomplete’ physically; disliking the scarring; leakages making people feel ‘useless’, knocking self‐confidence; feeling less able to be oneself because of the stoma; disclosing the presence of the stoma to others (friends/intimate partners); friends constantly referring to the stoma—perpetuating the sick role; perceived negative impact on friendships/worry that friends would not understand; loss of confidence and immediate post operative bodily debilitation impacted return to social participation—challenges re‐engaging with the world; disrupted romantic encounters/getting a partner; rejections/disgust/lack of support from intimate partners and family; stigma and taboo of stools; intimacy and sexual functioning an issue; additionally for gay men re removal of the rectum

Author(s), Year, Country Study title	i. Methodology ii. Sampling iii. Data capture iv. Data analysis	Sample: i. *N* = ii. Age (range) iii. Gender iv. Diagnosis v. Type of stoma (N) vi. Time with stoma (range)	Adjustment mechanisms/key issues extracted during data charting Realizations, personal attributes, attitudes, or aspects of personality that are perceived to help or hinder adjustment
Thorpe et al. 2016, UK^a^ Adjusting to bodily change following stoma formation: a phenomenological study	i. Longitudinal descriptive phenomenology ii. Purposive iii. individual interviews at three time points post‐operatively iv. Colaizzi, Van Manen IPA	i. 12 (3 lost to follow‐up) ii. 30's – 70's iii. M (6), F (6) iv. UC (2), CD (1), Rectal Cancer (5), Diverticulitis (4) v. Permanent colostomy (5); permanent ileostomy (1). Temporary colostomy (4). Temp Ileostomy (2) vi. 3–15 months	***Helpful factors:*** expecting improvement over time; familiarity with the stoma; accepting what cannot be changed; appliances better than historically, so easier to cope with; ability to project the same outward image post‐operatively, as pre‐operatively; being able to dress to hide the stoma; confidence, built over time, brings return to preferred ways of dressing; control over the stoma influenced by consistency and predictability of output, mastering stoma care, experiences of leakage. Takes time for all this to settle down post‐operatively—suggests 9 months; practical/specialist support and emotional readiness; having the right appliance that suits; time reduces dissonance between knowing and seeing bodily function but not being able to feel it; accepting the stoma as ‘part of me’; contrast between health and social life prior to surgery, and the freedom/wellness the stoma brings; transition from being cared for to self‐caring; older age range more optimistic that they would cope ***Hindrances*:** uncertainty over time it would take to get used to the stoma; changes in body appearance/shape of abdomen; appliance unsightly; avoiding looking at the stoma/appliance; stigma—concerns that others would see the stoma; having to wear different style of clothing—not ‘them’/ their choice; unpredictable stoma output; fear of flatus from stoma inhibits social activities; leakages, especially with no‐one to advise—compounds feelings of inadequacy and undermines confidence; dependence on others
Thorpe and McArthur, 2017^a^ UK Social adaptation following intestinal stoma formation in people living at home: a longitudinal phenomenological study	i. Longitudinal descriptive phenomenology ii. Purposive iii. individual interviews at three time points post‐operatively iv. Colaizzi, Van Manen IPA	Details as above	***Helpful factors***: positive contrast between pre‐surgery ill self, and post‐surgery improved physical functioning; self‐confidence comes competence managing the stoma; increased confidence, acceptance of, and familiarity with the stoma over time and being prepared (access to toilets, stoma care equipment ‘kit’, dietary manipulation) enables social participation and ability to cope with the stoma away from home; increased sense of control reduces self‐consciousness and stigma‐related concerns; positive support from intimate partners—not being alone with it all; resumption of sexual relationships; partners' preparedness to adjust and accommodate; supportive family and friends; humour; contact with others with a stoma; previous knowledge of stomas (in other people) helps set realistic expectations; access to stoma care nurse specialists and to supportive, helpful information; setting smaller achievable goals for social re‐integration and return to work; for men—being physically active ‘doing jobs around the house’; for women—contentedness with their clothed appearance; developing a new social normality that incorporates the stoma ***Hindrances***: perceived physical deterioration post‐surgery (especially if elective and quite well at the point of surgery); lengthy post‐op debilitation delays adjustment and return to social participation; lack of confidence in managing stoma away from home; a ‘misbehaving’ stoma; stigma—others being aware of the stoma; body image—change in shape of abdomen, choice of clothing; unsupportive partners; stigma and ‘othering’ from partners, family and friends; leakages; holding on to pre‐surgery ‘normality’; failure to achieve bodily mastery hampers individual's reconnection with their social worlds in the long‐term – home clung on to as ‘safe haven’
Mørkhagen and Nortvedt, 2023, Norway A Qualitative Study on How Younger Women Experience Living with an Ostomy	i. Descriptive and exploratory ii. Purposive iii. individual interviews carried out post‐operatively (*n* = 2) and pre and post change in stoma (*n* = 2) iv. Malterud's analysis method	Sample: i. *N* = 4 ii. 20–35 iii. F(4) iv. UC & CD v. Colostomy; ileostomy vi. 6–20	***Helpful factors:*** treated with dignity and empathy whilst recovering from surgery; follow up from nurses; providing information about stoa care and diet; being made aware of challenges in managing stoma; being informed of equipment available; having support from nursing staff with specific expertise in stoma care; living near a city or large hospital and closer to support; having time to prepare for surgery; having a temporary stoma fitted initially; symptom relief after surgery and greater control; time when stoma was formed ***Hindrances*:** lack of privacy and empathy in recovery; living in an area further from large hospital, resulting in less follow up support; limited discussions about sexuality and living with a stoma and fertility and living with a stoma; frequent leakage and/or ill fitting equipment; invisible condition and a lack of awareness from others

Abbreviations: CD, Crohn's disease; HP, hermeneutic phenomenology; IBD‐U, IBD unclassified; IPA, interpretive phenomenological analysis; RV, recto‐vaginal; UC, ulcerative colitis.

^a^ Thorpe et al. (2016) and Thorpe and McArthur (2017) report separate aspects arising from a single study with the same cohort of participants.

### 3.1 Descriptive and thematic analyses

Study dates, country of origin, title, methodological and sample (participant) details, and data relevant to those with IBD and experience of a stoma, were extracted from the 13 included studies (Allison et al., 2013; Danielsen, Soerensen, et al., 2013b; Horgan et al., 2020; Mørkhagen & Nortvedt, 2023; Morris & Leach, 2017; Nicholas et al., 2008; Persson & Hellström, 2002; Polidano et al., 2020; Savard & Woodgate, 2009; Sinclair, 2009; Smith et al., 2017; Thorpe et al., 2016; Thorpe & McArthur, 2017).

Extracted data were collated prior to thematic analysis (Braun & Clarke, 2006). Early similarities were identified by the senior author who assigned preliminary codes informed by the stated purpose, aims and objectives of the study. Following team discussion, core themes and sub‐themes were identified and agreed. Data charting enabled generation of themes running across all included papers, and identification of unique or rarely reported issues; the resulting rich data represented a range of experiences of people with IBD living with a stoma and the factors that influence adjustment. Four themes emerged: (1) facilitative factors; (2) barriers to adjustment; (3) personal attributes; and (4) time and temporality.

#### 3.1.1 Theme 1: facilitative factors

Factors that contributed positively to adjustment were identified across all included papers: *Preparedness and being informed*, *Tangible Benefits*, *Social and Clinical Support*, and *Disclosing the Stoma to Others*.

##### Preparedness and being informed

Often, stoma‐forming surgery became the only option for effectively managing symptoms (Horgan et al., 2020), but better preparedness led to more positive post‐operative responses. Planned surgery enabled patients to gather information, understand options and consider potential benefits (Allison et al., 2013; Mørkhagen & Nortvedt, 2023; Sinclair, 2009). The ‘directness’ of surgeons (Horgan et al., 2020) increased confidence in decision‐making. Information‐seeking reduced anxiety, enabled the ‘taking on’ of stoma self‐care, and increased patients' confidence in their ability to cope (Allison et al., 2013; Nicholas et al., 2008). Clinical nurse specialists, publications and websites were all helpful, but information‐seeking was more facilitative when led by the patient (Allison et al., 2013; Nicholas et al., 2008; Thorpe & McArthur, 2017). Having prior experience of ones' own temporary stoma (Sinclair, 2009) or witnessing one in others (Thorpe & McArthur, 2017) reduced anxiety.

##### Tangible benefits

Stoma surgery was viewed positively when it transformed patients' illness‐dominated lives (Allison et al., 2013; Horgan et al., 2020; Mørkhagen & Nortvedt, 2023; Morris & Leach, 2017; Polidano et al., 2020; Savard & Woodgate, 2009). In contrast, inability to perceive any benefits post‐surgery (Sinclair, 2009) or struggling with (normal, temporary) physical deterioration post‐operatively—especially if surgery was elective and the patient was well pre‐operatively (Smith et al., 2017)—obscured any potential benefits.

##### Social and clinical support

Support from family, friends and peers helped new ostomates of any age gain confidence with and adjust to life with their new stoma (Allison et al., 2013; Danielsen, Soerensen, et al., 2013b; Sinclair, 2009; Smith et al., 2017; Thorpe & McArthur, 2017). Parental support that enabled growing independence in adolescents and young adults as recovery progressed, was highly beneficial (Allison et al., 2013; Nicholas et al., 2008) whilst peer support and acceptance facilitated acceptance of new identity and reintegration into the young person's social world (Nicholas et al., 2008). Unconditional support from intimate partners was invaluable (Smith et al., 2017; Thorpe & McArthur, 2017); the partner's acceptance of the stoma, willingness to adjust and accommodate to meet new needs, and resumption of intimacy played a key role in strengthening ostomates' own sense of self‐worth and confidence (Smith et al., 2017; Thorpe & McArthur, 2017). Some gender variations were apparent—women wanted emotional support and reassurance (Allison et al., 2013) whilst men preferred practical support (managing the stoma) and to feel part of their peers' social world (Allison et al., 2013; Thorpe & McArthur, 2017).

Specialist clinicians played a key role in setting realistic expectations. Adjustment was facilitated when clinicians addressed issues that patients might experience post‐operatively honestly, rather than denying problems could arise (Danielsen, Soerensen, et al., 2013b; Horgan et al., 2020; Sinclair, 2009). Continued support from medical staff regardless of outcome (Allison et al., 2013), and ongoing access to a specified specialist stoma nurse before and after surgery (Allison et al., 2013; Danielsen, Soerensen, et al., 2013b; Horgan et al., 2020; Mørkhagen & Nortvedt, 2023; Sinclair, 2009; Thorpe et al., 2016) provided much‐needed practical and emotional support, and increased confidence in self‐management skills and adaptation.

Communicating, either in person or via the internet, with others who had undergone the same experience encouraged new ostomates to see that adjustment was possible (Danielsen, Soerensen, et al., 2013b; Nicholas et al., 2008; Sinclair, 2009; Thorpe & McArthur, 2017). Asking questions via the internet provided reassuring anonymity and a boldness to ‘basically ask anything’ (Nicholas et al., 2008).

##### Disclosing the stoma to others

Integration of the stoma into a new concept of the self was facilitated by purposeful disclosure of the stoma to others; whilst intentional visual reveals of the stoma pouch/appliance were rare, those who adjusted well‐shared information about their stoma with trusted people (Sinclair, 2009; Smith et al., 2017). Patients' self‐worth and confidence were reinforced when recipients of the information did not flinch at what patients often considered ‘a disgusting thing’, thus extending and strengthening the social support network.

#### 3.1.2 Theme 2: barriers to adjustment

Barriers to adjustment were more nuanced than simply being an absence or minimal presence of the facilitative factors reported above, and included: *Sudden and Unexpected Surgery*, *Poor Social and Clinical Support*, *Stigma and Body Image*, *Difficulties with the Stoma*, and *Self‐enforced Isolation and Restrictions*.

##### Sudden and unexpected surgery

The ability to accept and cope with a stoma was impaired when there was no time for physical or emotional preparation because surgery was sudden and unexpected (Horgan et al., 2020; Sinclair, 2009), previous repeated surgeries had led to minimal overall improvement in health status (Allison et al., 2013), and the possibility of needing a stoma was addressed very late in the illness trajectory (Horgan et al., 2020). Without time to assimilate/accept the need for stoma surgery, new ostomates felt emotionally unprepared for the stoma (Mørkhagen & Nortvedt, 2023; Polidano et al., 2020; Savard & Woodgate, 2009; Sinclair, 2009) and the need to care for it (Persson & Hellström, 2002; Savard & Woodgate, 2009; Sinclair, 2009).

##### Poor social and clinical support

The absence of appropriate social and clinical support to meet new ostomates' needs disrupted the development of self‐esteem and confidence that were necessary for adjustment; poor social support included overprotective parents and over‐vigilance in intimate relationships (Allison et al., 2013), unsupportive partners, family and/or friends (Smith et al., 2017; Thorpe et al., 2016), lack of access to age‐matched peers with the same experience (Horgan et al., 2020), and being treated as ‘Other’—being excluded from sexual intimacy by partners, and feeling rejected by family (Thorpe et al., 2016). Negative attitudes of non‐specialist ward staff immediately post‐operatively, including displaying disgust at the stoma and failing to appreciate that the experience was traumatic for patients, had an enduring negative effect on individuals, emotionally and relationally (Danielsen, Soerensen, et al., 2013b; Mørkhagen & Nortvedt, 2023; Sinclair, 2009). Hospital staff setting unrealistic expectations (particularly that recovery would be problem‐free) led to heightened anxieties and concerns when problems arose (Danielsen, Soerensen, et al., 2013b). Misinformation about what to expect, inadequate written information and poor pre‐ and post‐operative education hindered adjustment (Horgan et al., 2020; Sinclair, 2009). Being left to work it out alone, including locating reliable information online (Horgan et al., 2020), affected the ability to accept oneself as an ostomate (Thorpe & McArthur, 2017). When this poor support occurred alongside physical debilitation in the immediate post‐operative period, it led to a meaningful loss of confidence, eroded sense of self and heightened distress (Smith et al., 2017; Thorpe et al., 2016) with notable impact on the acceptance/adjustment trajectory.

##### Stigma and body image

New ostomates were often hampered by anticipated and perceived stigma; this convinced them that others would respond negatively, and prevented them sharing information about their new status and from accessing potential social support networks (Danielsen, Soerensen, et al., 2013b; Horgan et al., 2020; Nicholas et al., 2008; Persson & Hellström, 2002; Sinclair, 2009). New ostomates were anxious that the stoma may ‘reveal itself’ by being visible beneath their clothing (Savard & Woodgate, 2009; Sinclair, 2009; Thorpe et al., 2016; Thorpe & McArthur, 2017) and consequently, reluctantly moved away from their preferred type of clothing to maintain concealment (Savard & Woodgate, 2009; Thorpe et al., 2016). Changes in clothing and outward appearance could give a sense of control over the risk of unintended revealing (Horgan et al., 2020), but this concealment could also represent a disconnect from their embodied self and preferred presentation to others (Thorpe & McArthur, 2017). The unobtainable notion of bodily perfection pervading modern social commentary added a distressing pressure to those who felt that their now scarred and blemished body further distanced them from this ideal (Sinclair, 2009). Body image concerns affected genders differently; women were more concerned about surgical scars, the uncontrolled bowel activity that the stoma represents, and changes to abdominal shape and clothing choices (Allison et al., 2013; Thorpe et al., 2016; Thorpe & McArthur, 2017) whilst men worried about the impact of the stoma itself on their body image (Allison et al., 2013). Overcoming these concerns was necessary for adjustment to occur and could be achieved by balancing bodily changes with the overall benefits the stoma offered (Allison et al., 2013) and being able to project the same outward image post‐operatively, as pre‐operatively (Thorpe et al., 2016).

##### Difficulties with the stoma

The absence of stoma issues boosted confidence in the ability to manage, and therefore accept, the stoma (Polidano et al., 2020). In contrast, issues such as appliance leakage (Polidano et al., 2020; Savard & Woodgate, 2009; Sinclair, 2009; Thorpe et al., 2016; Thorpe & McArthur, 2017) and unpredictable stoma activity, noise and flatus (Thorpe et al., 2016), were deeply distressing. These experiences compounded feelings of inadequacy and undermined confidence, delaying acceptance and adjustment (Thorpe et al., 2016), heightening anxiety (Polidano et al., 2020), and causing some to regret having surgery (Savard & Woodgate, 2009). Some with a temporary stoma displayed an unwillingness to commit the emotional energy needed to adjust, preferring to ‘wait it out’ until stoma reversal (Polidano et al., 2020).

##### Self‐enforced isolation and restrictions

Stoma acceptance and willingness to join their social world as an ostomate seemed influential in adjusting to the new normal yet some, perhaps due to lack of confidence, avoided this re‐integration. Whilst unsurprising in the first few weeks post‐operatively, persistent self‐enforced isolation and social restrictions hampered adjustment due to separation from potential social support networks (Thorpe & McArthur, 2017). Some with a temporary stoma chose to restrict social life and dating until the stoma was reversed (Allison et al., 2013); others avoided social interactions, preferring to be alone because they felt (emotionally) safer (Persson & Hellström, 2002; Polidano et al., 2020) or did not feel ready to resume previous activities (Polidano et al., 2020).

#### 3.1.3 Theme three: personal attributes

Several attributes appear to influence the acceptance of and adjustment to a stoma. These are expressed as *Positive Expectations and Personality*, *Gender and Age*, and *Taking Control*.

##### Positive expectations and personality

Approaching stoma surgery and recovery expecting that ‘things would be better’ appeared beneficial. This expectation precipitated proactive steps (e.g., information seeking, social re‐integration), thus facilitating adjustment (Allison et al., 2013). Anticipating benefits from stoma surgery pre‐operatively (Nicholas et al., 2008), looking forward to enjoyable future activities (Polidano et al., 2020) and expecting things to improve with time even if the timespan was unknown (Thorpe et al., 2016; Thorpe & McArthur, 2017) were helpful adjustment factors. Personality was influential, as an internal locus of control (Rotter, 1966) appeared to enable positive thinking and actions (Nicholas et al., 2008). Those who expected positive recovery and reintegration made decisions and took actions to support that envisaged future such as setting smaller, more achievable goals for social and work re‐integration, thus building confidence up over time (Thorpe & McArthur, 2017). Self‐assessment of one's ability to cope (Allison et al., 2013) and characteristics such as determination, commitment, persistence, recognizing personal strengths and one's own role in adapting, taking direct action, having a positive attitude, accepting the stoma and what cannot be changed and ‘getting on with it’ all facilitated adjustment (Allison et al., 2013; Horgan et al., 2020; Nicholas et al., 2008; Polidano et al., 2020; Thorpe et al., 2016). In contrast, absence of these characteristics, yearning for the pre‐stoma body and life and being unwilling to let this go to integrate the stoma into the new life, was debilitating (Thorpe & McArthur, 2017).

##### Gender and age

Further to preferred types of support and body image issues reported above, gender and age were influential in other aspects of adjustment. For men, being physically active and being able to do jobs in the home facilitated adjustment, whilst women benefitted from feeling content with their clothed appearance (Thorpe & McArthur, 2017). Ostomates at different ages and stages of life required different levels of support, having different normal developmental psychosocial tasks to achieve alongside adjusting to their new status (Polidano et al., 2020; Sinclair, 2009); some young adults (aged 24–40 years) doubted their own ability to cope as an ostomate (Sinclair, 2009), whilst adolescents (aged 13–19 years) demonstrated remarkable adaptability, reflection, resilience and coping skills (Nicholas et al., 2008). Across two studies with age ranges of 23–72 years (Smith et al., 2017), and 30s – 70s (Thorpe & McArthur, 2017), older participants were more optimistic of their ability to cope with and integrate their stoma into ‘normal’ life.

##### Taking control

An internal locus of control (Braun & Clarke, 2006) means feeling in control and having influence over the things that affect one's life—taking responsibility for our actions and for the consequence of those actions. Accepting and adjusting well to life with a stoma was facilitated amongst those who took control and proactively worked to integrate the stoma into their new world. This included a growing sense of control and ability to plan (Persson & Hellström, 2002) through increasing competence, confidence and independent stoma management (Horgan et al., 2020; Nicholas et al., 2008) having the right appliance to suit (Thorpe et al., 2016), choosing and being happy with concealing clothing (Horgan et al., 2020), and accepting that ‘bad days’ would happen (Nicholas et al., 2008). Accepting the stoma (Horgan et al., 2020) and a growing feeling of emotional control about being an ostomate (Horgan et al., 2020; Thorpe et al., 2016) further aided adjustment. Transitioning from being cared for, to becoming self‐caring, added to the sense of control (Thorpe et al., 2016; Thorpe & McArthur, 2017) but was not always straightforward (Sinclair, 2009).

Realizing and embracing rather than resisting the changes that were needed to live well with the stoma (Polidano et al., 2020) and to overcome practical challenges such as appliance leakages (Nicholas et al., 2008; Polidano et al., 2020) aided the sense of control. Taking control enabled ostomates to move forward; being prepared to manage the stoma away from home and setting personal goals for returning to work and travel facilitated re‐integration into their social world (Thorpe & McArthur, 2017).

##### Time and temporality

Time (as measured objectively by a clock) and temporality (subjective progression through moments) play out differently, perhaps partially explaining individual variances in trajectory from dependence, to confidence, independence and mastery of new life with the stoma. The emotional and psychological move towards acceptance occurred at different rates for different people (Mørkhagen & Nortvedt, 2023; Nicholas et al., 2008) creating complex relationships between the many factors already addressed in this review. All included studies referred to these concepts of time and temporality, from the time it took to discuss options and accept the need for surgery (Allison et al., 2013), to not addressing the possibility of stoma surgery until very late in the illness trajectory (Horgan et al., 2020), to the timing of the actual surgery (Allison et al., 2013; Mørkhagen & Nortvedt, 2023), and acknowledging that adjustment takes time (Morris & Leach, 2017; Nicholas et al., 2008; Polidano et al., 2020; Savard & Woodgate, 2009; Sinclair, 2009; Smith et al., 2017; Thorpe et al., 2016; Thorpe & McArthur, 2017). Adjustment was inexorably linked to confidence, self‐esteem, and a sense of practical and emotional control over the stoma—all of which took time to develop and was different for everyone (Thorpe et al., 2016). Time was needed to grieve (for the lost body) and acknowledge (if not accept) the new stoma (Nicholas et al., 2008); those shocked by the sight of the stoma or who struggled to overcome loss of confidence and immediate physical debilitation after surgery (Sinclair, 2009) or who endured negative responses from ward staff (Danielsen, Soerensen, et al., 2013b; Sinclair, 2009), felt the repercussions for many months, if not years, afterwards. In contrast, those who experienced a greater positive contrast between their pre‐and post‐stoma self were more tolerant of the changes and adjusted with greater ease and speed than others for whom the contrast was less pronounced (Morris & Leach, 2017). ‘*The story of living with a chronic disease* (such as IBD) *is intimately connected to the story of living with an ileostomy*’ (Sinclair, 2009) and thus has implications on the time and process of adjustment following stoma‐forming surgery.

## 4 DISCUSSION

This scoping review pools the available qualitative evidence on learning to live with a stoma for IBD, highlighting the influential clinical, health‐related, personal and psychosocial factors. It demonstrates that adjustment/acceptance are complex processes requiring much more than practical competence in stoma management.

Specialist stoma nurses educate patients to achieve the competent stoma management that is essential for enabling post‐operative hospital discharge; these review findings demonstrate that patients also need emotional support from these specialist nurses, available for as long as is necessary. Insights into decision‐making around stoma‐forming surgery also support our earlier work (Dibley, Czuber‐Dochan, et al., 2018), reinforcing the message that patients need time to assimilate information, and accept the need for stoma surgery and prepare themselves for it; they will likely cope better post‐operatively and have a smoother transition to living well with their stoma if given time to prepare mentally for the experience. A similar observation is reported in quantitative QoL studies—outcomes are better when the intervention (internal ileo‐anal pouch, or stoma formation) matches patient preference (Kuruvilla et al., 2012) and when disease status is improved (Abdalla et al., 2016). Awareness of the long‐term damaging psychological effects of negative reactions to a patient's new stoma by non‐specialist nurses can guide better education and support of these nurses by the specialist clinical team.

Currently in the UK, approximately 600 stoma nurse specialists provide crucial support to +/− 21,000 new ostomates annually (Coloplast, 2019). National variations in provision of specialist stoma care nursing services may impact on availability, yet patients need to be able to self‐refer via multiple methods of access if issues with their stoma arise (Coloplast, 2019). Further education and support may be achieved with contact with laypersons (Danielsen, Soerensen, et al., 2013c) or stoma ‘buddies’ (Dibley, Czuber‐Dochan, et al., 2018) who themselves have experience of a stoma and can appreciate first‐hand the complex relational and emotional aspects that exist.

None of the 13 included studies refer specifically to parastomal hernias which are responsible for up to 57% of all stoma‐related complications in Crohn's disease (Aboulian, 2019) and hamper patients' abilities to gain control over stoma function. Control is influenced by consistency and predictability of output, mastering stoma care, and experiences of leakage—yet, there are indications that it takes on average nine months (not the three months traditionally cited) (Thorpe et al., 2016) for these usual post‐operative issues to settle down. This review demonstrates that ongoing emotional and psychosocial adjustment beyond nine months is also influenced by numerous other social, personal and psychosocial aspects factors, including the availability of social support, perceptions of stigma, concerns about body image, personality (including locus of control), perceived benefits of surgery, gender and age. However, we still do not know how that influence is exerted, why individuals respond very differently to stoma‐formation or whether there is any relationship between these factors and the adjustment trajectory to living well with the new stoma.

In earlier work, we identified that prior exposure to those with a stoma, personality, response to dirt/disgust trait, resilience, childhood socialization towards bodily functions, and willingness/ability to seek out relevant information prior to surgery all influence how people with IBD respond to stoma‐forming surgery (Dibley, Norton, & Whitehead, 2018). These, and the factors confirmed in this review could contribute to development of an assessment tool to initially assess the likely response to stoma‐forming surgery and identify those more likely to need additional pre‐and post‐operative support, and to map the patient's post‐operative adjustment trajectory over time. We concur with Thorpe and colleagues (Thorpe et al., 2016; Thorpe & McArthur, 2017) over the need for more longitudinal research into adjustment to a stoma. They collected data at three time points following surgery (three, six and 15 months) [33.34] but a longer time frame would allow for deeper exploration and understanding of the nuances and complexities of adjustment. Employing ‘*a longitudinal approach* (would) *capture the process leading up to the achievement of biographical renewal*’ (Polidano et al., 2020, p. 355). Ten of the 13 included studies are cross‐sectional, and report *that* adjustment is or is not achieved, relying on participant recall of potentially influential factors. The QUALITATIVE relationship between psychological well‐being and stoma surgery has been explored (Dibley, Czuber‐Dochan et al., 2018), but again, this is cross‐sectional and investigates the issue retrospectively. Resilience in IBD has been investigated in a prospective longitudinal cohort study, to identify why some people thrive despite the considerable challenges IBD presents, whilst others struggle to cope (Sirois & Hirsch, 2017). Similarly, mapping adjustment to a new stoma in real time over a longer time span would give greater insight into the process of adjustment, and the complex aspects which play a part in integrating the stoma into a person's life or in disrupting this process. It has been suggested that mixed cohorts should be avoided in cross‐sectional studies since factors including the type of stoma (ileostomy or colostomy, temporary or permanent) can influence patient experiences (Smith et al., 2017); however, we propose that multi‐cohort longitudinal studies would support collection and comparison of data from different age, disease, and stoma‐type cohorts, enabling between‐cohort comparisons. This would facilitate identification of adjustment aspects that are common to all patients experiencing a new stoma, and those that are specific to disease, stoma type, and age groups—such as whether the biographical renewal described elsewhere (Polidano et al., 2020) is more difficult for older people when identity and self‐perception are already set, or for younger people in whom identity is still being established. Longitudinal research would also enable the mapping of average trajectories according to different patient profiles; in combination with the new assessment tool proposed above, this would facilitate effective identification and ongoing assessment of patient need, from the moment the possibility of stoma surgery is first suggested.

The final stage of the scoping review framework (Levac et al., 2010) recommends ongoing consultation with stakeholders. We aim to build on the robust Patient and Public Involvement and Engagement (PPIE) in our preliminary work (Dibley, Czuber‐Dochan, et al., 2018) and in this review, to develop and deliver the new assessment tool and the longitudinal research outlined above.

### 4.1 Strengths and limitations

This review is the first to map available qualitative evidence on factors influencing adjustment to living well with a stoma for people with IBD. It has followed a recommended process, assuring thoroughness and credibility in the results. We attempted to extract IBD‐specific data from mixed cohort studies, but cannot guarantee precision in doing so. Further, by excluding the word ‘ostomy’ (and derivatives) from our search strategy, American publications may have been overlooked; however, we did locate international publications, enhancing the global relevance of this review.

## 5 CONCLUSION

This scoping review demonstrates that adjustment to stoma surgery for IBD is a complex process influenced by numerous organizational, health‐related, personal and time‐related factors. Since the evidence is primarily cross‐sectional and adjustment is a process, there is clear need for longitudinal work to explore the complex relationships between, and influences of, personal factors, and to demonstrate trajectories of adjustment. Knowledge of influential factors can usefully inform the development of a new assessment tool to facilitate identification of each patient's personal resources and likely response to a stoma, monitor adjustment over time and enable individualized support strategies to be implemented.

### REGISTRATION

The protocol for this review is registered with the Open Science Framework (DOI: https://doi.org/10.17605/OSF.IO/QRE9T).

## AUTHOR CONTRIBUTIONS

**Lesley Dibley** was involved in conceptualization and methodology. **Ryan Essex and Lesley Dibley** were involved in data curation and writing—original draft. **Lesley Booth, Fuschia Sirois and Jennie Burch** were involved in writing—reviewing and editing.

## FUNDING INFORMATION

No funding was received for carrying out this review.

## CONFLICT OF INTEREST STATEMENT

The authors declare no conflicts of interest.

### PEER REVIEW

The peer review history for this article is available at https://www.webofscience.com/api/gateway/wos/peer‐review/10.1111/jan.16254.

## Supporting information


**Data S1:** Supporting Information

**File S1:** xxx.

## Data Availability

This paper did not generate any new data, the data that was synthesised is publicly available.
