# Supplementary material for: A scoping review of the qualitative literature reporting experiences of living with a stoma for inflammatory bowel disease
Source: J Adv Nurs. 2024 May 23;81(1):53–68. doi: 10.1111/jan.16254 (PMC11638519; doi:10.1111/jan.16254)
Supplement: Supplementary file 2 — File S1: xxx. [file JAN-81-53-s002.docx]

**Supplementary file 1**

Below we report a more detailed methodology related to our search strategy, eligibility criteria and data extraction.

**Stage 1: Identifying the Research Question**

When developing the research question the concept (personal factors), target population (people with a stoma for IBD) and outcomes of interest (psychosocial adjustment and/or quality of life) were considered [17]. The scoping review question therefore was: *What is known about any personal psychosocial and quality of life factors that inform adjustment to living well with an intestinal stoma for IBD*?

### **Stage 2: Identifying Relevant Studies**

As per the guidance [17] the team agreed the scope of the study. Search terms and search strategy were developed to capture the core concepts, related to our population of interest and their post-stoma surgery psychosocial or quality of life outcomes, reported in qualitative literature [Table S1].

Table S1 : Search terms and strategy

| **Focus of paper** | **Search terms** |
| --- | --- |
| Quality of life and/or psychosocial wellbeing | psychosocial OR "psycho social" OR psycho-social OR psycholog* OR social OR "quality of life" OR QOL OR HRQOL OR "health related quality of life" OR "health-related quality of life" |
| Intervention | stoma OR ileostomy OR colostomy |
| Patient group/condition | IBD OR "inflammatory bowel disease" OR Crohn* OR "ulcerative colitis" |
| Research type | qualitative |

Searches were undertaken on 07/08/23 using Scopus, Web of Science, CINAHL, Medline and PsycInfo. The reference lists of included papers were also searched for relevant articles.

**Stage 3: Study selection**

Following removal of duplicates, the titles and abstracts were scanned by both the first author (RE) and senior author (LD) and articles were rejected if there was no qualitative component, or the focus was not on IBD. The selected articles were then read through in full by the senior author, screened against the full inclusion/exclusion criteria [Table S2], and verified by the first author.

Table S2: Inclusion and exclusion criteria

| **Inclusion criteria** | **Exclusion criteria** |
| --- | --- |
| Original research | Opinion pieces, editorials, literature reviews, abstracts only, conference proceedings |
| Published in English |  |
| Published since 2000 reflecting modern medical, surgical, and nursing techniques |  |
| Qualitative, or mixed-methods design with extractable qualitative data |  |
| Adult participants (aged 18 years and over) with a diagnosis of IBD |  |
| In mixed cohort studies, IBD patient data reported separately |  |
| Key search terms present in title and/or abstract |  |

**Stage 4: Charting the Data**

As per the guiding methodology [18], the team determined *a priori* the variables to extract to describe the included studies and answer the scoping review question. Although the option existed, no revisions to the form were needed. Data extracted included author demographics, study aim/purpose, study design, participant demographics (age, gender), type of stoma, time spent living with a stoma, and adjustment mechanisms, defined as ‘realisations, personal attributes, attitudes or aspects of personality that are perceived to help or hinder adjustment’. The first and senior authors charted data from all included studies, whilst the rest of the team worked on a smaller number of included studies each, thus ensuring that each study was charted several times. Data charting was carried out by all authors independently, before discussing and reaching consensus. Consistency in the charted data evidenced the credibility, robustness and completeness of the process.

**Collating, Summarising and Reporting the Results**The scoping review methodology [18] recommends addressing this stage in three distinct steps of 1) descriptive and thematic analyses; 2) reporting results and relating these to the research question; and 3) considering the relationship between findings, study purpose and discussing implications. These sub-stages reflect the traditional reporting framework of Results, Discussion, and Conclusions and Recommendations (or Relevance to Clinical Practice), as presented below. Study characteristics are described below in a descriptive narrative; thematic analysis of the extracted data identified four core themes.

**Ethics**

No ethical approval was required for this scoping review which did not collect original data from human participants. Ethical permissions for the included studies were appropriately obtained by the original authors.
